# Sleep architecture in idiopathic hypersomnia: the influence of age, sex, and body mass index

**DOI:** 10.1038/s41598-024-67203-6

**Published:** 2024-07-16

**Authors:** Anne-Sophie Deshaies-Rugama, Samantha Mombelli, Hélène Blais, Zoran Sekerovic, MiaClaude Massicotte, Cynthia Thompson, Milan Nigam, Julie Carrier, Alex Desautels, Jacques Montplaisir, Nadia Gosselin

**Affiliations:** 1 Center for Advanced Research in Sleep Medicine, Research Center of the Centre Intégré Universitaire de Santé et de Services Sociaux du Nord de l’Île-de-Montréal, Montreal, Canada; 2 Department of Psychology, Université de Montréal, Montreal, Canada; 3 Department of Neuroscience, Université de Montréal, Montreal, Canada; 4 Department of Psychiatry and Addictology, Université de Montréal, Montréal, Canada; 5 Center for Advanced Research in Sleep Medicine, Hôpital du Sacré-Cœur de Montréal, 5400 Boul. Gouin Ouest, Office J-5135, Montréal, Québec H4J 1C5 Canada

**Keywords:** Hypersomnolence, Excessive daytime sleepiness, Polysomnography, Sleep disorders, Sleep architecture, Sleep disorders, Diagnostic markers

## Abstract

This study aimed to progress the understanding of idiopathic hypersomnia (IH) by assessing the moderating influence of individual characteristics, such as age, sex, and body mass index (BMI) on sleep architecture. In this retrospective study, 76 IH participants (38.1 ± 11.3 years; 40 women) underwent a clinical interview, an in-laboratory polysomnography with a maximal 9-h time in bed and a multiple sleep latency test (MSLT). They were compared to 106 healthy controls (38.1 ± 14.1 years; 60 women). Multiple regressions were used to assess moderating influence of age, sex, and BMI on sleep variables. We used correlations to assess whether sleep variables were associated with Epworth Sleepiness Scale scores and mean sleep onset latency on the MSLT in IH participants. Compared to controls, IH participants had shorter sleep latency (p = 0.002), longer total sleep time (p < 0.001), more time spent in N2 sleep (p = 0.008), and showed trends for a higher sleep efficiency (p = 0.023) and more time spent in rapid eye movement (REM) sleep (p = 0.022). No significant moderating influence of age, sex, or BMI was found. More severe self-reported sleepiness in IH patients was correlated with shorter REM sleep latency and less N1 sleep in terms of proportion and duration (ps < 0.01). This study shows that, when compared to healthy controls, patients with IH had no anomalies in their sleep architecture that can explain their excessive daytime sleepiness. Moreover, there is no moderating influence of age, sex, and BMI, suggesting that the absence of major group differences is relatively robust.

## Introduction

Idiopathic hypersomnia (IH) is characterized by excessive daytime sleepiness (EDS), irrepressible need to sleep during the daytime and/or prolonged nocturnal sleep duration. Patients can also report long and non-restorative naps as well as sleep inertia or sleep drunkenness^1–3^. The diagnosis procedure includes a clinical interview, an in-laboratory overnight polysomnography (PSG), a Multiple Sleep Latency Test (MSLT) and/or an objective measure of long sleep duration with ad-lib PSG or actigraphy^4^. Objective markers of IH are long sleep duration (≥ 660 min per 24 h) or abnormal mean sleep latency on MSLT (≤ 8 min), although controversies exist regarding these criteria^5^. Diagnosis challenges are mainly due to the absence of specific biomarkers or phenotypic characteristics. Moreover, the pathophysiology of IH is yet to be understood. Due to the severity of EDS, patients report significant impacts and a reduced quality of life, including difficulties driving, challenges at work and school, and more limited family and social activities^6,7^. Generally, IH is considered a rare condition, leading to the majority of studies focusing on relatively small patient cohorts, typically comprising fewer than 20 individuals^8^. The estimated prevalence of IH ranged from 0.002% to 0.01%^9–12^ based on studies conducted between 1997 and 2012. However, in a study conducted in 2024, the potential public health implications of this sleep disorder were highlighted. The findings revealed that IH symptoms were present in up to 1.5% of the Wisconsin Sleep cohort, suggesting a higher prevalence in the general population than previously estimated^13^.

A few studies have explored the nocturnal PSG sleep variable specificities of IH to better understand the symptomatology reported by patients. According to a meta-analysis by Plante^8^ that included 10 studies with a total of 249 patients, IH is associated with longer total sleep duration, shorter sleep latency, lower percentage of N3 sleep, and higher percentage of rapid eye movement (REM) sleep when compared to healthy controls. However, this meta-analysis also highlighted the need to further investigate IH sleep architecture. Indeed, among the 10 studies included in the meta-analysis, 7 assessed 20 or fewer IH patients, while 6 had undefined diagnostic criteria or criteria based on a previous classification (e.g., International Classification of Sleep Disorders, 2nd edition)^14^.

An aspect to consider when investigating sleep architecture in IH is whether and how patient characteristics, namely age, sex, and body mass index (BMI), could modify sleep architecture markers of IH. In fact, the meta-analysis described above reported a lower sleep efficiency among IH patients compared to controls, particularly in women^8^. Regarding BMI, a study of 75 IH patients showed that those with longer sleep duration (633 ± 76 min) had lower BMI and were younger compared to IH patients with shorter sleep duration (517 ± 60 min)^15^. However, in a study including 37 patients with IH and 79 patients with subjective IH (defined as not having objectively long sleep duration or abnormal MSLT), it was observed that among the subset of patients with clear-cut and probable IH, those exceeding 19 h of sleep on 32-h protocol were more frequently found to be overweight, compared to those who slept fewer than 19 h^16^. Furthermore, the onset of IH symptoms is often observed between 10 and 30 years old^17^, and one study reported an average age of 30 years old at diagnosis^1^. Taken together, these studies highlight some unanswered questions. Firstly, whether age at diagnosis leads to different IH subtypes is unknown but could orient pathogenesis hypotheses. Secondly, regarding sex-differences, IH is more prevalent in women^18^, but whether they differ from men in terms of phenotype characteristics is not clear^17^. Finally, being overweight or obese has been reported among IH patients^17^, and presenting these comorbidities associated with IH could also be specific to a subphenotype or pathophysiologic origin. Hence, it is necessary to conduct additional research using sufficiently large sample sizes to investigate the moderating impact of age, sex, and BMI on the group effects identified in sleep architecture within studies on IH. This exploration will help provide a comprehensive understanding of the role of these factors in shaping sleep patterns and characteristics in IH patients.

A significant challenge related to IH research is that 15 to 25% of patients have significant depressive symptoms^19^ and therefore may use antidepressants, including selective serotonin and norepinephrine reuptake inhibitors (SSNRIs) and bupropion, which inhibits norepinephrine and dopamine reuptakes. Many patients cannot stop antidepressant use for the PSG recording. Excluding them could reduce the generalizability of the results by ruling out a subgroup of IH patients. On the other hand, including them would introduce a bias in the study of sleep architecture (i.e., REM sleep suppression). Therefore, documenting medication use and analyzing patients with and without antidepressant use could represent the best option, as previously performed in patients with traumatic brain injury^20^. To our knowledge, there is no documentation as to the specific effects of SSNRIs on sleep architecture in the IH population.

Lastly, it remains unclear whether abnormal nighttime sleep architecture can account for EDS. In a study of 266 participants with complaints of hypersomnolence (Epworth Sleepiness Scale [ESS] > 10) from diverse etiologies, those with short sleep latency (MSLT ≤ 8 min, 49% of the sample) had comparable sleep architecture, but presented higher sleep efficiency on PSG than participants with normal MSLT (> 8 min)^21^. The 96 patients of this study with total sleep time ≥ 19 h during a 32-h bed rest protocol^16^ and a MSLT ≤ 8 min were younger, presented a higher sleep efficiency and a trend for higher slow wave sleep proportion compared to other hypersomnolent participants with no objective markers of long sleep duration and/or abnormal MSLT. These results suggest that patients presenting EDS and objective markers of IH have a more consolidated sleep and a trend for a deeper sleep compared to those who do not present these objectives markers. We still do not know, however, if EDS severity is associated with abnormal PSG variables in IH patients.

The present study aimed at characterizing the sleep architecture of IH patients in comparison to healthy controls using a large sample, and to evaluate the moderating influence of age, sex, and BMI on group differences. Moreover, although the main analyses were performed on participants who did not use psychoactive medication, supplementary analyses were performed in IH patients using antidepressants to verify whether they constitute a specific subgroup in terms of sleep architecture. Finally, we explored whether changes in sleep architecture were associated with self-reported and objective markers of EDS severity.

## Methods

### Participants

This retrospective study included participants with IH who were selected from the Center for Advanced Research in Sleep Medicine (CARSM) of the *Centre intégré universitaire de santé et de services sociaux (CIUSSS) du Nord-de-l’Île-de-Montréal* clinical database. They were all referred to the sleep clinic due to a complaint of EDS, which had been present for at least three months prior to testing. They were recorded with a nighttime PSG with a maximal 9-h time-in-bed window, followed by an adapted MSLT (i.e., 4 naps) at the sleep clinic between 2001 and 2019. All participants received a diagnosis of IH confirmed by a sleep medicine specialist, based on results from their PSG, MSLT, and a clinical interview. Medical records were reviewed based on the following eligibility criteria: (1) age between 18 and 60 years old at the time of testing; (2) confirmed diagnosis of IH by a sleep physician and according to the International Classification of Sleep Disorders 3rd edition of the American Academy of Sleep Medicine^4^; (3) at least 6 h of sleep during the nighttime PSG; (4) a MSLT $\le$ 8 min; and (5) less than two sleep onsets in REM sleep period in the MSLT and PSG procedures. Based on the medical chart review, patients were excluded if they had : (1) a change of sleep disorder diagnosis over time; (2) an apnea–hypopnea index $\ge$ 15; (3) a diagnosed psychiatric condition (except depression and anxiety), neurological or sleep disorder other than IH (e.g., restless legs syndrome, sleepwalking, etc.); (4) use of antipsychotic and anticonvulsant medication; (5) shift work leading to an atypical sleep schedule; (6) circadian rhythm disorders, chronic sleep deprivation or any other condition that could explain the EDS complaint; and (7) psychostimulant medication use that was not stopped at least 5 half-lives before the PSG. Patients using antidepressants and who were not able to stop their medication before the PSG were excluded from the main analyses. Their medication use was documented, and these participants were considered in supplementary analyses. Based on those criteria, 76 IH patients without medication (med- group) were included and 46 IH patients with antidepressant medication (med+ group) were added for supplementary analyses only. While the participants in this study originate from the same database as a previous investigation conducted by our team^22^, there was substantial variations in inclusion and exclusion criteria. The present study's primary objective was to characterize the nighttime sleep macroarchitecture of objective IH, while the previous article aimed at exploring differences between patients with objective vs. subjective IH with a focus on self-reported unrefreshing naps.

A group of 106 healthy control subjects aged 18 to 60 years old were selected from two local databanks: 9 from The Montreal Archive of Sleep Studies (MASS)^23^ and 97 from Carrier’s lab databank^24–27^. These subjects were all tested at the CARSM between 1999 and 2013. Exclusion criteria were the same as for participants with IH, in addition to: (8) use of any medication affecting PSG (including antidepressants); (9) diagnosis of any psychiatric or sleep disorder; (10) Beck Depression Inventory-Short Form (BDI-SF)^28^ score > 4 or Beck Depression Inventory-II (BDI-II)^29^ score > 13. The *CIUSSS du Nord-de-l’Île-de-Montréal* Research Ethics Board approved the study protocol (#2020–1905), and all experiments were performed in accordance with their relevant guidelines and regulations. All control participants gave their informed consent.

### Data acquisition

#### Questionnaires

Questionnaires were administered to IH participants, mostly the night before the PSG recording, to document sleepiness and mood symptoms: The Epworth Sleepiness Scale^30^, the BDI-II^29^ and the BAI^31^. 12 healthy subjects completed the BDI-II, 73 completed the BAI, and 58 completed the BDI-SF^28^.

#### Polysomnography

Bedtime and waketime were determined according to the participant’s usual sleep schedule. Participants had to minimally self-report a stable sleep schedule of at least 6 h, and/or show stable sleep in sleep diaries or actigraphy in the week before the PSG recording. For the in-laboratory PSG, IH patients and controls were not allowed to go to bed before 22:00 and to wake up after 7:00, thus spending a maximum of 9 h in bed. Polysomnographic recordings were acquired with a Grass system using Harmonie software (Stellate Systems, Montreal, Canada, https://stellate-harmonie.software.informer.com/) or a Natus system (Brain Monitor, Trex, and Embla NDx) (Natus Medical Incorporated, Middleton, USA, https://natus.com/neuro/natus-brain-monitor-eeg-amplifier/; https://natus.com/neuro/natus-embla-ndx-psg-amplifier/). The PSG montage included at least four EEG derivations (C3, C4, O1 and O2) referred to earlobes with a sampling rate of 128 Hz for participants tested before 2013, and of 256 Hz for participants tested after 2013 (amplification factor of 10 000; bandpass 0.3–100 Hz). An electrooculogram, an electrocardiogram and a chin electromyogram were also used for the night recording. A surface electromyogram on both anterior tibialis muscles was included to measure periodic leg movements. Respiration monitoring was conducted utilizing an abdominal strain gauge and an oronasal cannula for some patients between 2000 and 2005, while others underwent monitoring with a nasal thermistor. Oxygen saturation was measured with a transcutaneous finger pulse oximeter.

#### Mean sleep latency test

The MSLT was carried out for the IH participants only. IH patients underwent an adapted version of the MSLT with four naps^32^. Participants were offered the opportunity to take four naps two hours apart (9:00, 11:00, 13:00 and 15:00 or 10:00, 12:00, 14:00 and 16:00), the day after the standardized PSG procedure. The set-up for measuring sleep latency consisted only of the EEG with the same four electrodes (C3, C4, O1 and O2), as well as the electrooculogram and the chin electromyogram. All other sensors were removed in the morning following the PSG. The average nap sleep onset latency was calculated. Total sleep time for each nap as well as the number of sleep onsets in REM periods were also measured.

### Sleep macroarchitecture analyses

A trained sleep technologist identified sleep stages and sleep events from PSG and MSLT recordings according to the most recent criteria, using 30-s epochs^32,33^. Respiratory events were determined according to standard criteria^33,34^. Apneas were considered as a reduction of airflow of ≥ 90% for at least 10 s, and hypopneas as a diminution of airflow of ≥ 30% for at least 10 s, ending with an oxygen desaturation ≥ 3% or an arousal. The following variables were determined: sleep latency, REM latency, time in bed, total sleep time, sleep efficiency, wake after sleep onset (WASO), and duration and percentage of each sleep stage (N1, N2, N3 and REM). Apnea–hypopnea index (AHI), micro-arousal index and periodic leg movements of sleep (PLMS) index associated or not with arousals were also measured.

### Statistical analyses

Group comparisons on demographic, clinical and sleep architecture characteristics were performed using Mann–Whitney U tests or Kruskal–Wallis tests for quantitative data. Chi-square tests were used for nominal variables. Non-parametric tests were used as the assumption of normality was not met for most quantitative data. When the assumption of normality was not met for quantitative data, logarithmic transformations in base 10 were performed to normalize distributions as much as possible. When significant group differences were observed in Kruskall-Wallis tests, pairwise comparisons with Dunn’s tests and Bonferroni corrections for multiple comparisons were used for post-hoc comparisons.

To explore the role of age, sex, and BMI on IH sleep architecture, moderation analyses were conducted in SPSS with PROCESS macro version 4.2^35^. Age, sex, and BMI were used as moderator variables (in three different analyses) and the independent variable was the group (IH or controls) for each sleep architecture variable. Age, sex, and BMI were used as covariates when they were not the moderator factors in those analyses. To assess whether self-reported and objective markers of EDS were associated with sleep architecture in IH, partial correlations corrected for age, sex, and BMI were performed. Supplementary analyses compared IH patients using antidepressant medication that could not be stopped for the PSG, to the 76 IH patients without medication and 106 controls. Kruskall-Wallis tests were used for quantitative data, while chi-square tests were used for nominal variables. We used SPSS Statistics version 27 (IBM Corp., 2020, https://www.ibm.com/products/spss-statistics) for all statistical analyses. To account for multiple analyses and comparisons, the level of statistical significance was set at p < 0.01 for all analyses. P values between 0.01 and 0.05 are described as trends.

## Results

### Sample characteristics

Demographic, clinical and questionnaire data, as well as statistics for IH patients (excluding those using antidepressants) and control participants are displayed in Table 1. No group differences were found for age and sex. Yet, IH patients had higher BMI (U = 4899.5, p < 0.001), they showed a trend towards more depression (U = 459.0, p = 0.047) and had more anxiety symptoms (U = 3332.0, p < 0.001) compared to healthy controls. 27 (36%) IH patients in our sample were overweight (25 ≤ BMI < 30), 11 (15%) were clinically obese (BMI > 30), 6 had clinical depression (suggested cut-off at 23 on the BDI-II)^36^ and 10 had clinical anxiety (suggested cut-off at 16 on the BAI)^31^.

**Table 1:** Demographic and clinical characteristics of IH and control participants.

	Controls (n = 106)	IH (n = 76)	U or ${\varvec{\chi}}$^2^ values	p values	Effect sizes η^2^ or Cramer’s V
Age, years	38.1 ± 14.1	38.1 ± 11.3	4018.5	0.978	–
Sex, nb. of females (%)	60 (56%)	40 (53%)	0.282	0.595	–
Body Mass Index, kg/m^2^	23.7 ± 3.7	25.8 ± 4.2; n = 72	4899.5	** < 0.001**	**0.058**
Epworth Sleepiness Scale Scores	NA	16.5 ± 3.4	NA	NA	–
Beck Depression Inventory Scores	6.2 ± 4.8; n = 12	11.4 ± 8.2; n = 56	459.00	0.047	0.058
Beck Depression Inventory – Short Form Scores	0.8 ± 2.0; n = 58	NA	NA	NA	-
Beck Anxiety Inventory Scores	2.0 ± 3.1; n = 73	8.0 ± 7.3; n = 56	3332.00	** < 0.001**	**0.290**
Mean Sleep Latency on MSLT, min	NA	4.8 ± 1.7	NA	NA	–

Data are expressed as mean $\pm$ standard deviation.

Significant values are in bold.

*IH* idiopathic hypersomnia, *MSLT* mean sleep latency test, *NA* not applicable or not available, *nb* number, *min* minutes, *kg* kilograms, *m* meter.

### Group differences on sleep architecture

**Figure 1:**
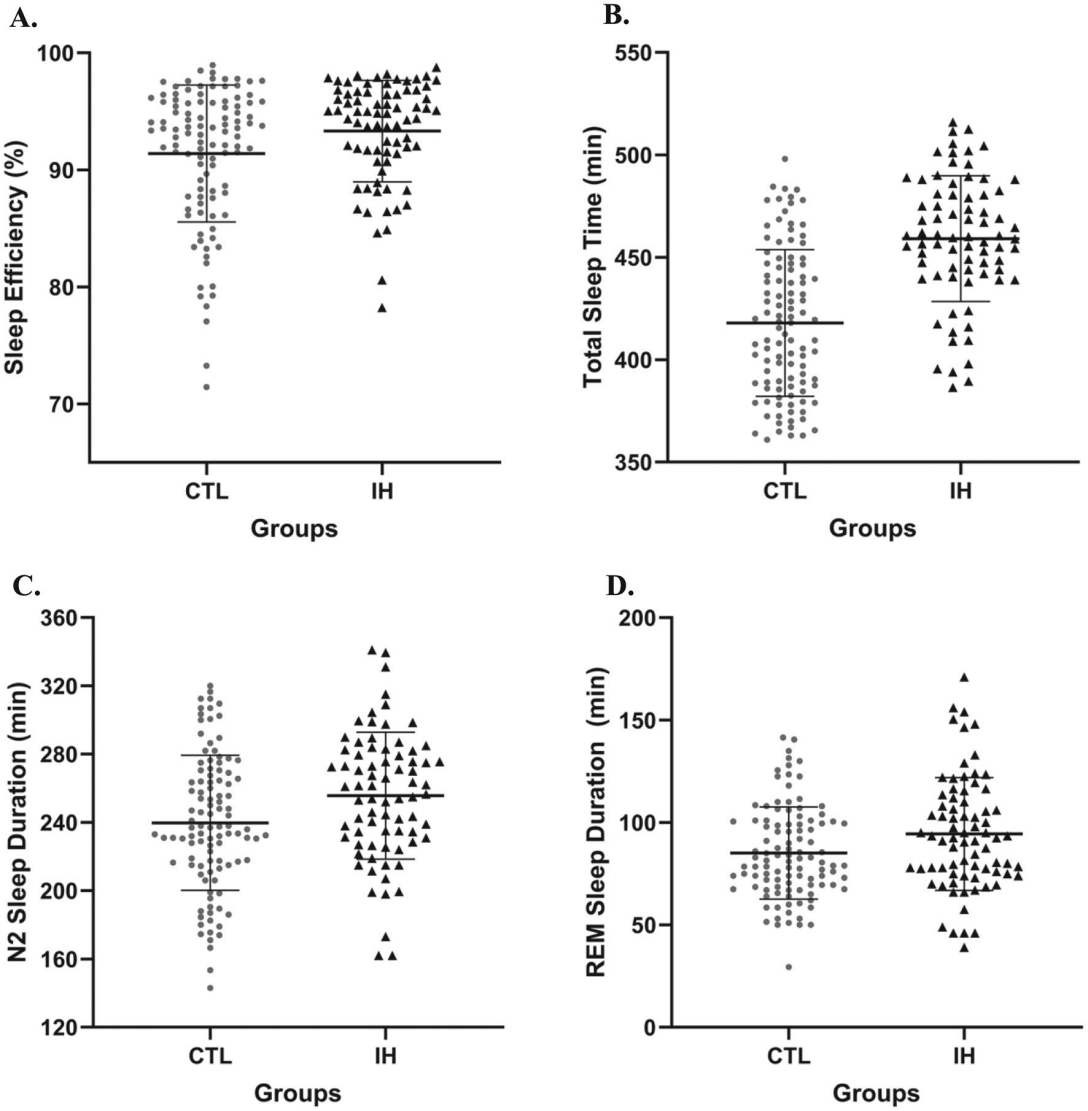
Individual data point distributions for controls (CTL, represented by grey circles) and participants with idiopathic hypersomnia (IH, represented by dark triangles). Each plot includes the mean (indicated by a horizontal line) and standard deviation (represented by whiskers); (**A**) All-night sleep efficiency (%); (**B**) Total sleep time (minutes); (**C**) Total N2 sleep duration (minutes); (**D**) Total REM sleep duration (minutes).

Table 2 presents PSG variables for IH patients (excluding those using antidepressants) and control participants as well as statistics and effect sizes. Compared to healthy controls, IH patients had significantly longer time in bed (U = 6155.0, p < 0.001), longer total sleep time (U = 6468.5, p < 0.001), shorter sleep onset latency (U = 2928.0, p = 0.002), and a trend for higher sleep efficiency for the nighttime PSG (U = 4822.0, p = 0.023). Proportion of IH patients and controls with high sleep efficiency was comparable, as 79% of IH patients and 70% of healthy subjects had a sleep efficiency of 90% or higher (${\varvec{\chi}}$^2^_(1, 182)_ = 1.903, p = 0.168). Compared to controls, IH patients spent significantly more time in N2 sleep when measured in minutes (U = 4964.0, p = 0.008) and a similar trend was observed for REM sleep (U = 4829.0, p = 0.022), but no group effect was found for sleep stage proportions when measured in percentage of total sleep time. The IH group had significantly higher AHI (U = 5507.5, p < 0.001) and PLMS index (U = 5480.0, p < 0.001) than healthy subjects. Sixteen IH participants (21%) had a PLMS index > 15, while 12 controls (11%) had a PLMS index > 15. Fifteen IH participants (20%) had an AHI > 5, and 10 controls (9%) had an AHI > 5. Figure 1 presents individual datapoints for IH and control participants for PSG variables with significant group differences or trends for group differences.

**Table 2:** PSG macroarchitecture variables for IH and Control groups.

	Controls (n = 106)	IH (n = 76)	U values	p values	Effect sizes η^2^
Total sleep time, min	418.0 ± 35.8	459.3 ± 30.6	6468.5	** < 0.001**	**0.266**
Time in bed, min	472.2 ± 35.7	502.6 ± 21.3	6155.0	** < 0.001**	**0.202**
Sleep onset latency, min	10.2 ± 8.6	7.4 ± 7.3	2928.0	**0.002**	**0.054**
REM latency, min	92.0 ± 42.6	89.6 ± 45.9	3795.5	0.507	–
WASO, min	39.9 ± 28.7	32.4 ± 20.9	3368.5	0.110	–
Sleep efficiency, %	91.4 ± 5.6	93.3 ± 4.3	4822.0	0.023	0.028
Stage N1 sleep, min	39.1 ± 17.3	45.7 ± 27.1	4336.0	0.379	–
Stage N2 sleep, min	239.8 $\pm$ 39.6	255.6 ± 37.1	4964.0	**0.008**	**0.039**
Stage N3 sleep, min	54.0 ± 37.4	63.4 ± 35.9	4701.5	0.055	–
REM sleep, min	85.1 ± 22.5	94.4 ± 27.5	4829.0	0.022	0.029
Stage N1 sleep, %	9.4 ± 4.1	10.1 ± 6.4	3904.0	0.724	–
Stage N2 sleep, %	57.3 ± 7.4	55.7 ± 7.6	3482.0	0.119	–
Stage N3 sleep, %	13.1 ± 9.2	13.7 ± 7.7	4377.5	0.319	–
REM sleep, %	20.2 ± 4.5	20.5 ± 5.4	3984.0	0.900	–
Micro-arousal index, nb/h	8.3 ± 4.4	9.7 ± 6.1	4482.0	0.195	–
Apnea–hypopnea index, nb/h	1.5 ± 2.8	2.7 ± 3.2	5507.5	** < 0.001**	**0.098**
Periodic limb movements index, nb/h	6.0 ± 11.0	10.0 ± 11.7	5480.0	** < 0.001**	**0.094**

Data are expressed as mean $\pm$ one standard deviation.

Significant values are in bold.

*IH* idiopathic hypersomnia, *REM* rapid eye movement, *WASO* wake after sleep onset, *min* minutes, *nb* number, *h* hour.

### Influence of age, sex, and BMI on sleep architecture in patients with IH

Regression models were conducted to assess the interaction between Group and age, sex, or BMI on sleep architecture. Regression models were adjusted for age, sex, and BMI when these variables were not employed as the moderation factor in the analysis. The results showed no significant Group X age, sex, or BMI interaction on sleep variables.

### Correlations between ESS scores, MSLT and sleep architecture in patients with IH

**Figure 2:**
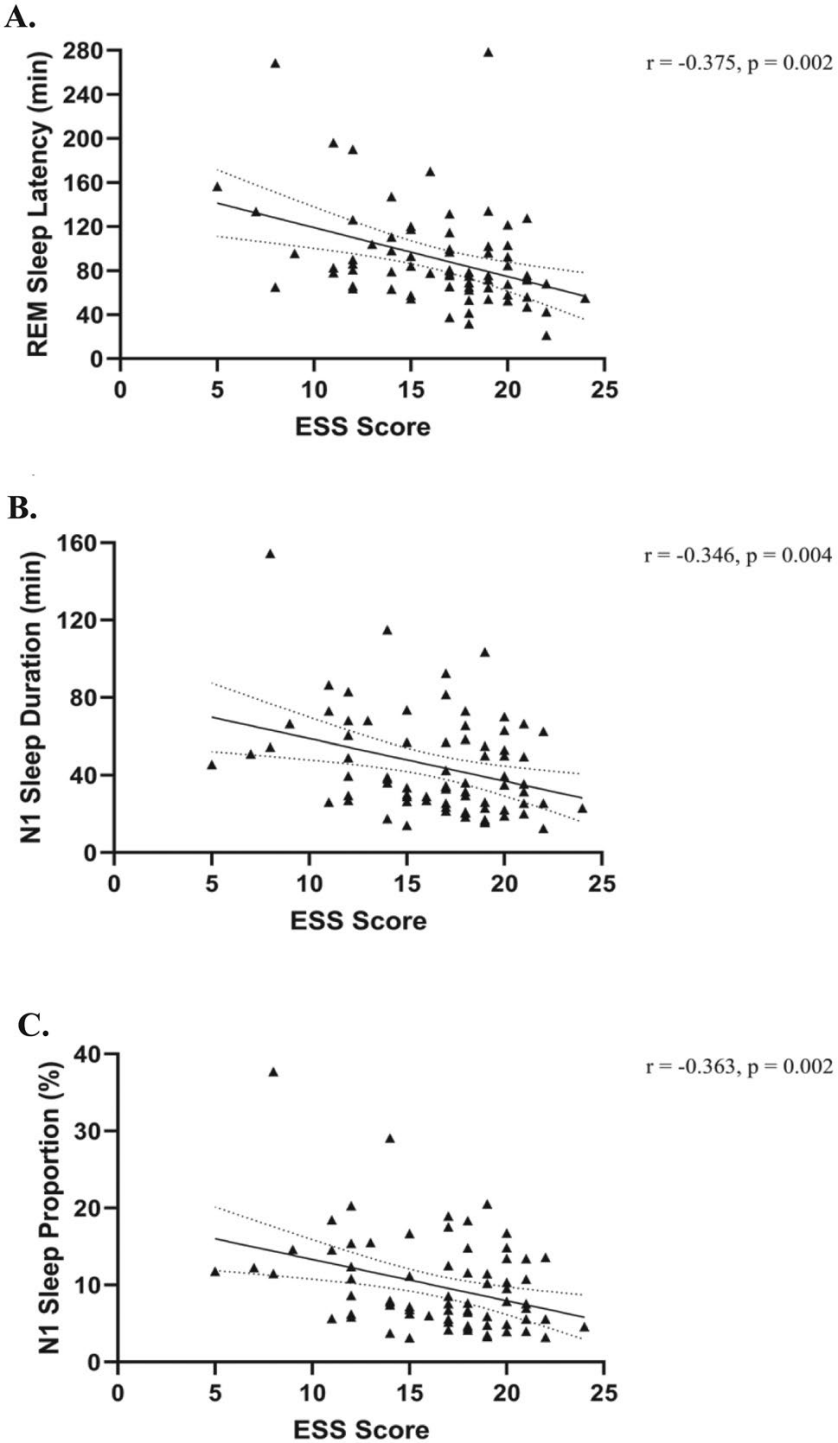
Partial correlations (solid lines) with 95% confidence intervals (dotted lines) between subjective EDS symptoms and sleep architecture variables corrected for age, sex, and BMI in participants with IH. Graphs display the individual data point distributions. (**A**) higher ESS scores correlated with shorter REM sleep latencies (minutes); (**B**) higher ESS scores correlated with shorter N1 sleep durations (minutes); (**C**) higher ESS scores correlated with smaller N1 sleep proportions (%).

Figure 2 shows the relationship between objective and subjective EDS and sleep architecture for 72 IH patients, with correlations corrected for age, sex, and BMI. All correlations can be found in Table 3. Figure 2 panels depict negative associations between ESS scores and REM sleep latency, time spent in N1 sleep and percentage of N1 sleep stage (ps < 0.01). Moreover, trends for correlations were observed between higher ESS scores and shorter nighttime sleep onset latencies (p = 0.03), higher sleep efficiency (p = 0.04), and smaller micro-arousal index (p = 0.019). Globally, more severe self-reported EDS was associated with a more continuous sleep. No significant correlations were found between MSLT and PSG variables, as shown in Table 3.

**Table 3:** Partial correlations corrected for age, sex and BMI between ESS score, MSLT and sleep variables in 72 IH patients.

	ESS Score	MSLT
Total sleep time, min	r = 0.177, p = 0.146	r = − 0.199, p = 0.101
Time in bed, min	r = 0.100, p = 0.413	r = − 0.022, p = 0.855
Sleep onset latency, min	r = − 0.255, p = 0.034	r = 0.211, p = 0.082
REM latency, min	**r = − 0.375, p = 0.002**	r = − 0.041, p = 0.737
WASO, min	r = − 0.210, p = 0.083	r = 0.012, p = 0.924
Sleep efficiency, %	r = 0.244, p = 0.044	r = − 0.031, p = 0.799
Stage N1 sleep, min	**r = − 0.346, p = 0.004**	r = − 0.145, p = 0.233
Stage N2 sleep, min	r = 0.115, p = 0.347	r = − 0.065, p = 0.598
Stage N3 sleep, min	r = 0.201, p = 0.098	r = − 0.024, p = 0.848
REM sleep, min	r = 0.134, p = 0.273	r = 0.025, p = 0.839
Stage N1 sleep, %	**r = − 0.363, p = 0.002**	r = − 0.123, p = 0.313
Stage N2 sleep, %	r = 0.049, p = 0.691	r = 0.045, p = 0.714
Stage N3 sleep, %	r = 0.174, p = 0.153	r = 0.003, p = 0.980
REM sleep, %	r = 0.117, p = 0.339	r = 0.066, p = 0.592
Micro-arousal index, nb/h	r = − 0.281, p = 0.019	r = 0.067, p = 0.586
Apnea–hypopnea index, nb/h	r = 0.054, p = 0.660	r = − 0.145, p = 0.234
Periodic limb movements index, nb/h	r = − 0.218, p = 0.072	r = 0.018, p = 0.886

Significant values are in bold.

*ESS* Epworth Sleepiness Scale, *MSLT* Mean Sleep Latency Test, *WASO* wake after sleep onset, *REM* rapid eye movement, *min* minutes, *nb* number, *h* hour.

### Supplementary analyses with IH patients using antidepressant medications

Table S1 displays demographic and clinical characteristics of IH patients using antidepressant medications that cannot be stopped for the PSG (med+) compared to the other IH patients (med-) and controls. The med+ group included more women (80%) than the med- IH group (52%), (${\varvec{\chi}}$^2^_(1, 122)_ = 9.515, p = 0.002). They mainly used selective serotonin reuptake inhibitors (SSRIs, 87% of the group). No differences between the two IH groups were found for depression, anxiety, and sleepiness symptoms on questionnaires, and they did not differ on the MSLT. Table S2 presents group differences on PSG variables. The med+ group significantly differed from controls by having longer total sleep time, time in bed, REM sleep latency, and N1 sleep duration (all ps < 0.01), as well as having significantly higher micro-arousal, apnea–hypopnea and PLMS indices (all ps < 0.001).

Compared to the med- group, the med+ group had longer sleep onset latency (p < 0.001), REM sleep latency (p < 0.001), more minutes of N1 sleep (p = 0.008) and higher micro-arousal (p < 0.001) and PLMS indices (p < 0.001). The two IH groups did not differ on sleep efficiency, WASO duration, and sleep stage percentages.

### Sensitivity analyses

Considering the group differences found on the PLMS index and the AHI, we performed sensitivity analyses between these indices and sleep architecture variables in IH and control participants separately. More specifically, moderation analyses were conducted to assess the Group X AHI and the Group X PLMS interactions on sleep architecture, with age, sex, and BMI as covariates. No interactions between Group X AHI or Group X PLMS index or main effects of AHI or PLMS index were observed in relation to any sleep variables, suggesting that PLMS index and AHI cannot explain the results found for sleep architecture in the present study.

## Discussion

This study aimed at characterizing the sleep architecture of IH patients in comparison to healthy controls using a large sample, and to explore the influence of age, sex, BMI, and antidepressant medication on sleep variables. We found that IH subjects had longer total sleep time (even if we used a maximal 9-h sleep window) and shorter sleep onset latency compared to control participants. Although no group differences were found on sleep stage proportions, IH participants spent more time in N2 and they tended to have more time spent in REM sleep. Interestingly, despite having higher AHI and PLMS index compared to controls, IH patients showed a trend towards a higher sleep efficiency. As for the moderating influence of age, sex, and BMI on groups, no significant impact was found on sleep variables. Several PSG variables correlated with more severe self-reported EDS, namely shorter REM sleep latency and smaller N1 sleep duration and proportion. However, there was no significant relationship between PSG nocturnal sleep variables and the MSLT. IH participants using antidepressant medication had longer sleep onset latency and REM sleep latency, more minutes in N1 sleep and higher micro-arousal and PLMS indices than IH participants not using antidepressant medication.

Overall, this study did not show any PSG abnormalities that could explain the EDS observed in patients with IH. Instead, we noticed the opposite pattern: patients with more severe symptoms had less N1 sleep and tended to sleep more continuously. Unlike earlier studies, no influence of age, sex, and BMI was found on sleep parameters, suggesting that these observations are relatively robust among persons with IH.

### IH patients have no PSG abnormalities that can explain their EDS

In the present study, IH participants, who slept for longer durations than healthy controls, spent more time in N2 and a trend was found towards longer time spent in REM sleep. However, sleep stage proportions did not differ between patients with IH and healthy controls. According to a meta-analysis published in 2018^8^, IH patients showed a higher REM sleep proportion and a lower combined N2 and N3 sleep proportion compared to healthy controls. Similarly, a study published the same year that compared 37 patients with IH to 21 controls reported a lower N2 sleep proportion and a higher REM sleep proportion in IH as compared to healthy controls^16^. The discrepancies between those results and the present study could be explained by smaller sample sizes in previous studies, participant characteristics (e.g., IH severity, IH with vs. without long sleep duration), or the methodology used (e.g., studies with restricted vs. unrestricted time in bed). Taken together, the present and previous studies suggest that no clear and consistent abnormality in sleep stage proportion emerge in patients with IH, especially when they are restricted to a time- in-bed window (9 h in the present paper).

The present study showed that IH patients have an adequately consolidated sleep (with a trend towards higher sleep efficiency) compared to controls, despite having more sleep disruptors such as breathing disturbances and PLMS. Interestingly, correlation and moderation analyses showed no Group X AHI or Group X PLMS index interactions on sleep variables. Therefore, the sleep continuity of both IH patients and controls remained unaffected by the degree of sleep disruptors (i.e., AHI and PLMS index). It would be worthwhile to pay close attention to the level of arousability among IH patients in future studies. For example, protocols where auditory stimuli are presented during sleep could be performed to test arousal thresholds of IH patients.

Better sleep consolidation (e.g., lower N1 sleep, trends for higher sleep efficiency) in IH was found in patients not taking medication as well as in those using antidepressants at the time of PSG recording when compared to healthy controls. The observed sleep efficiency result lacks consistency across the existing literature. For example, there was no group differences between controls and IH patients on sleep efficiency in most studies included in the meta-analysis by Plante, but a sex-specific difference was observed where IH women had reduced sleep efficiency compared to control women^8^. One of the studies included in that meta-analysis reported a significantly lower sleep efficiency in 14 IH compared to 14 healthy subjects^37^. On the other hand, another study reported that IH patients had significantly higher sleep efficiency than controls^16^, which is in line with our findings. This inconsistency may be due, in part, to differences in IH diagnosis criteria used, sample sizes, undetected type-2 narcolepsy with higher sleep fragmentation and/or presence of IH subtypes. Regarding subtypes, another study did not find a significant difference between IH and controls on sleep efficiency, but found that IH patients with long total sleep time (> 600 min of nighttime sleep) had significantly higher sleep efficiency than IH patients without long sleep time^15^. In the present study, 79% of IH patients had a sleep efficiency > 90%, compared to 70% of healthy controls. This high sleep efficiency is incompatible with the proposed hypothesis suggesting that symptomatology in IH is attributable to sleep fragmentation^38^. While observations of consolidated sleep are typically linked to healthy sleep patterns within the general population^39^, in the case of IH patients, such consolidated sleep appears to be associated with self-reported sleep inertia, sleep drunkenness and EDS^40^. Therefore, it appears that individuals with IH exhibit a similar ability to maintain sleep compared to controls, despite experiencing notable EDS. This contradicts conventional notions regarding EDS being primarily linked to disrupted or fragmented nocturnal sleep. Further exploration of the sleep architecture in IH, such as investigating slow wave activity, is necessary to determine whether a heightened sleep drive and/or an unusually diminished wakefulness drive could elucidate EDS in IH despite a trend for more continuous sleep (i.e., higher sleep efficiency).

### Limited moderating influence of age, sex, and BMI on sleep architecture

Our findings do not support the notion that specific individual characteristics have an influence on the sleep architecture of IH, as age, sex and BMI did not moderate the effects of groups on PSG parameters. However, one study has previously reported a moderating role of age on sleep architecture. More specifically, Vernet and Arnulf^15^ found that IH patients with long sleep time were younger and had higher sleep efficiency than those with normal sleep time. Unfortunately, in the present study, we did not have access to ad-lib PSG and we were not able to classify our participants as presenting or not long sleep duration. It is possible that IH patients who are diagnosed at a younger age have more pronounced deep or consolidated sleep compared to those diagnosed at an older age. It is also possible that group differences on sleep architecture tend to disappear with age. Longitudinal studies will be needed to better understand the effect of age on IH sleep architecture.

The present study did not confirm the sex differences on IH sleep efficiency reported previously^8^. One previous study showed that IH patients with long sleep time were more overweight than IH patients without long sleep time^16^. The prevalence of overweight among patients with IH compared to the general population remains uncertain. However, a meta-analysis revealed a higher prevalence of obesity among patients with central hypersomnolence disorders (specifically type-1 narcolepsy)^41^, yet the precise mechanisms underlying this weight gain are still not understood^42^. Particular attention could be paid to overweight or obese IH patients and the possibility of a change in diagnosis over time in this subgroup.

Hence, according to our findings, no distinct phenotype appears to emerge from age, sex, or BMI for IH patients. Nonetheless, given the variety in clinical manifestations of IH—such as prolonged and non-restorative naps, cognitive fog, sleep inertia, and symptoms of depression^40^— the existence of phenotypes rooted in pathophysiology could be conceivable. This conjecture is supported by the heterogeneous nature of literature findings on sleep architecture and the challenge of differential diagnosis^43^.

### Self-reported EDS, but not MSLT results, correlates with PSG variables

Characterizing the relationship between self-reported and objective markers of EDS and sleep variables in individuals with IH has the potential to shed light on the underlying mechanisms contributing to their symptoms. Indeed, if IH participants have poor sleep quality, such as fragmented sleep or short sleep time, stronger EDS symptoms would be expected. However, in the present study, IH patients presenting stronger EDS symptomatology tended to have a more consolidated sleep, as shown with a trend to a higher sleep efficiency, smaller micro-arousal index and significantly smaller proportion of N1 sleep. We could hypothesize that IH participants have a high propension to sleep despite a tendency of having better sleep quality and normal to long sleep duration. However, there is an absence of significant association between MSLT results and nighttime sleep architecture in IH. Prior studies have indicated that MSLT and ESS scores assess distinct aspects of sleepiness. Our results support previous findings suggesting a poor validity of the MSLT to capture EDS in some central hypersomnolence disorders, such as IH^44,45^, as it is the gold standard for physiological sleep propensity but does not exhibit the strongest correlation with morbidity associated to sleep disorders^46^. Johns^47^ even found that the MSLT is the least accurate test for capturing sleepiness in everyday life, with the ESS demonstrating superior precision, which seems to be the case in our cohort. It is worth noting that MSLT might be insufficiently sensitive to represent EDS in IH due to its poor test–retest reliability^48^. To our knowledge, results of the present study are the first directly correlating IH nocturnal PSG sleep characteristics to objective and subjective markers of EDS. Notwithstanding, a recent study using a group comparison approach revealed that among hypersomnolent participants undergoing a 32-h bed rest protocol, those with longer total sleep time during the bed rest period (≥ 19 h) exhibited shorter latencies on the MSLT and higher sleep efficiency on nocturnal PSG in comparison to those without extended bed rest sleep duration^21^. Longer total sleep time during the bed rest period (≥ 19 h) accompanied by objective EDS (MSLT ≤ 8 min) was also associated with higher sleep efficiency participants without extended bed rest sleep duration. Severe EDS is therefore associated with a trend to more consolidated sleep, which is in line with the hypothesis that IH patients may have abnormally weak wakefulness drive or stronger sleep pressure compared to healthy subjects.

### Effects of antidepressant medication on IH sleep architecture

To date, there is a notable absence of studies examining the specific impact of antidepressant medication on sleep architecture in patients with IH, despite the well-established comorbidity rate of up to 25% between IH and depressive symptoms^19,49^. Patients are therefore often tested on PSG while using antidepressant medication. In the present study, we observed a minimal influence of medication on nocturnal sleep architecture in individuals with IH. Analyses indicated that IH participants who were taking antidepressants did not exhibit significant differences in the main sleep variables compared to other IH participants, except for a prolonged sleep onset latency, REM sleep latency and N1 sleep duration. Those results were expected as SSNRIs are known to delay REM sleep latency and increase N1 sleep duration in subjects without sleep disorders^50,51^. Our results also showed that IH patients using antidepressants had higher micro-arousal and PLMS indices than both controls and other IH participants, which can indicate a more fragmented sleep, which is concordant with observations that SSNRIs can impair sleep continuity and increase PLMS^50,52^. Since IH participants using antidepressants were mainly women, those results are in line with the findings of Plante^8^, where women with IH had worse sleep efficiency compared to healthy women. Future studies should offer a detailed description of psychoactive medication use and perform distinct statistical analyses in patients using and not using psychoactive medication, as this approach facilitates between study comparisons, prevents the exclusion of a patient subgroup, and increases results generalizability.

### Limitations

First, since our IH participants underwent a clinical protocol for assessing IH, subjects could not sleep ad-libitum like in 24 h and bed-rest protocols^15,16,53^. Therefore, their total sleep time might have been underestimated due to the sleep schedule necessary for clinical assessment and MSLT the next day. Participants also did not have a habituation night, which is recommended by the American Academy of Sleep Medicine^4^ to avoid the first-night effect, and to evaluate a sleep structure that is more representative of their typical sleep patterns^54,55^. Moreover, PSG were collected using two different recording systems, which could have introduced some bias in the computed variables. On the other hand, all scoring underwent review by the same senior sleep technologist according to the most recent criteria, and participants underwent PSG recordings with identical setups in the same laboratory, reducing the potential for bias. Finally, our sample was based on IH and healthy control participants whose data were collected over 20 years. IH and control participants thus came from different protocols and were not assessed with the same anxiety and depression symptom questionnaires. It is noteworthy that most questionnaires were completed the evening preceding the PSG; however, it is conceivable that some participants completed them in the morning after the PSG. Although this data was not available to us, it is important to consider that the timing of completion could potentially impact some results. Since the present study was conducted retrospectively and included participants from different protocols, some healthy subjects had been screened for depression and anxiety before undergoing the in-laboratory PSG (e.g., maximum scores on BDI-SF ≤ 4 and BDI-II ≤ 13). Furthermore, control participants did not undergo measurement of their EDS symptoms using MSLT or ESS; however, none reported any complaints of EDS. The results pertaining to those variables thus need to be considered carefully.

## Conclusion and clinical implications

The results of the present study contribute to the understanding of the sleep macroarchitecture characteristics found in IH, which could help in distinguishing it from other central hypersomnolence disorders. Knowing that IH patients have good sleep continuity can help differentiate IH and narcolepsy (especially narcolepsy type-2), which is characterized by more fragmented sleep than in controls^56–58^. Future studies should explore more specific sleep structures in IH, for instance sleep stage transitions and its microstructure, to assess if specific abnormalities could help us better understand this idiopathic disorder.

### Supplementary Information

Supplementary Tables.

## Data Availability

The datasets generated during and/or analysed during the current study are available from the corresponding author on reasonable request.
