# Supplementary material for: Sleep architecture in idiopathic hypersomnia: the influence of age, sex, and body mass index
Source: Sci Rep. 2024 Jul 16;14:16407. doi: 10.1038/s41598-024-67203-6 (PMC11252996; doi:10.1038/s41598-024-67203-6)
Supplement: Supplementary file 1 — Supplementary Tables. [file 41598_2024_67203_MOESM1_ESM.docx]

**Sleep architecture in idiopathic hypersomnia: the influence of age, sex, and body mass index**

Anne-Sophie Deshaies-Rugama, B.Sc.,^1,2^ Samantha Mombelli, M.Sc.,^1,4^ Hélène Blais, B.Sc.,^1^ Zoran Sekerovic, M.Sc.,^1^ MiaClaude Massicotte, B.Sc.,^1,2^ Cynthia Thompson, Ph.D.,^1^ Milan Nigam, M.D.,^1,3^ Julie Carrier, Ph.D.,^1,2^ Alex Desautels, M.D., Ph.D.,^1,3^ Jacques Montplaisir, M.D., Ph.D.,^1,4^ Nadia Gosselin*, Ph.D.^1,2^

1. Center for Advanced Research in Sleep Medicine, Research center of the Centre intégré universitaire de santé et de services sociaux du Nord de l’Île-de-Montréal, Montreal, Canada
2. Department of Psychology, Université de Montréal, Montreal, Canada
3. Department of Neuroscience, Université de Montréal, Montreal, Canada
4. Department of Psychiatry and Addictology, Université de Montréal, Montréal, Canada

Submitted to: Scientific Reports

Date of resubmission: June 3^rd^, 2024

Corresponding author:

Nadia Gosselin, Ph.D.

Center for Advanced Research in Sleep Medicine

Hôpital du Sacré-Cœur de Montréal

5400 boul. Gouin Ouest, office J-5135, Montréal, Québec, H4J 1C5, Canada

Tel: 514-338-2222 ext. 7717; Fax: 514-338-3893

Email: nadia.gosselin@umontreal.ca

|  | Controls (A)  (n=106) | IH med- (B)  (n=76) | IH med+ (C)*  (n=46) | H or $\boldsymbol{\chi}$^2^values | p values | Group differences | Effect sizes  η^2^ or Cramer’s V |
| --- | --- | --- | --- | --- | --- | --- | --- |
| Age, years | 38.1 ± 14.1 | 38.1 ± 11.3 | 37.2 ± 10.6 | 0.141 | 0.932 | - | - |
| Sex, nb. of women (%) | 60 (56%) | 41 (53%) | 37 (80%) | 10.239 | **0.006** | **A, B** $\boldsymbol{>}$**C** | **0.212** |
| Body Mass Index, kg/m^2^ | 23.7 ± 3.7; n =105 | 25.8 ± 4.2; n =72 | 27.5 ± 5.6; n =43 | 19.828 | **<0.001** | **A < B, C** | **0.082** |
| Epworth Sleepiness Scale Scores | - | 16.5 ± 4.0; n =75 | 16.8 ± 4.0 | 0.177 | 0.674 | - | - |
| Beck Depression Inventory Scores | 6.2 ± 4.8; n =12 | 11.4 ± 8.2; n =56 | 17.4 ± 11.6 n =21 | 10.235 | **0.006** | **A < C** | **0.096** |
| Beck Depression Inventory – Short Form Scores | 0.8 ± 2.0; n = 58 | - | - | - | **-** | - | - |
| Beck Anxiety Inventory Scores | 2.0 ± 3.1; n =73 | 8.0 ± 7.3; n =56 | 11.9 ± 9.4; n =21 | 49.725 | **<0.001** | **A < B, C** | **0.325** |
| Mean Sleep Latency on MSLT, min | - | 4.7 ± 1.7 | 5.1 ± 1.7 | 1.725 | 0.189 | - | - |

**Supplementary Table 1.** Demographic and clinical characteristics of IH participants without medication, IH patients using antidepressants, and control participants

Data are expressed as mean $\pm$ standard deviation. IH = Idiopathic hypersomnia; med+ = use of antidepressant medication during PSG recordings; med- = no use of antidepressant medication during PSG recordings; MSLT = Mean Sleep Latency Test, nb = number, min = minutes, kg = kilograms, m=meter.

**Supplementary Table 2.** PSG Macro-architecture in participants with idiopathic hypersomnia using or not using antidepressant medication, and control participants

|  | Controls (A)  (n=106) | IH med- (B)  (n=76) | IH med+ (C)*  (n=46) | H values | $\boldsymbol{\rho}$ | Group differences | | Effect sizes  η^2^ |
| --- | --- | --- | --- | --- | --- | --- | --- | --- |
| Total sleep time, min | 417.9 ± 35.7 | 459.3 ± 30.6 | 454.9 ± 31.2 | 58.245 | **< 0.001** | | **A < B, C** | **0.250** |
| Time in bed, min | 472.2 ± 35.7 | 502.6 ± 21.3 | 500.2 ± 37.6 | 44.304 | **< 0.001** | | **A < B, C** | **0.188** |
| Sleep onset latency, min | 10.2 ± 8.6 | 7.4 ± 7.3 | 11.0 ± 7.4 | 16.595 | **<0.001** | | **B < A, C** | **0.065** |
| REM latency, min | 92.0 ± 42.6 | 89.6 ± 45.9 | 153.1 ± 88.9; n = 44 | 27.404 | **<0.001** | | **A, B < C** | **0.114** |
| WASO, min | 39.9 ± 28.7 | 32.4 ± 20.9 | 29.6 ± 20.7 | 5.620 | 0.060 | | **-** | **-** |
| Sleep efficiency, % | 91.4 ± 5.9 | 93.3 ± 4.3 | 93.2 ± 5.5 | 7.415 | 0.025 | | A < B, C | 0.024 |
| Stage N1 sleep, min | 39.1 ± 17.3 | 45.7 ± 27.1 | 52.8 ± 25.2 | 9.771 | **0.008** | | **A, B < C** | **0.035** |
| Stage N2 sleep, min | 239.8 ± 39.6 | 255.6 ± 37.1 | 247.0 ± 46.4 | 6.989 | 0.030 | | A < B | 0.022 |
| Stage N3 sleep, min | 54.0 ± 37.4 | 63.4 ± 35.9 | 71.5 ± 39.7 | 7.973 | 0.019 | | A < B, C | 0.027 |
| REM sleep, min | 85.1 ± 22.5 | 94.4 ± 27.5 | 83.6 ± 41.4 | 4.807 | 0.090 | | - | - |
| Stage N1 sleep, % | 9.4 ± 4.1 | 10.1 ± 6.4 | 11.8 ± 6.1 | 5.205 | 0.074 | | - | - |
| Stage N2 sleep, % | 57.3 ± 7.4 | 55.7 ± 7.6 | 54.2 ± 8.6 | 6.047 | 0.049 | | A > C | 0.018 |
| Stage N3 sleep, % | 13.1 ± 9.2 | 13.7 ± 7.7 | 15.9 ± 9.5 | 3.463 | 0.177 | | - | - |
| REM sleep, % | 20.2 ± 4.5 | 20.5 ± 5.4 | 18.1 ± 8.8 | 1.908 | 0.385 | | - | - |
| Micro-arousal index, nb/h | 8.3 ± 4.4 | 9.7 ± 6.1 | 12.4 ± 6.5 | 15.774 | **< 0.001** | | **A, B < C** | **0.061** |
| Apnea-hypopnea index, nb/h | 1.5 ± 2.8 | 2.7 ± 3.2 | 2.9 ± 3.0 | 29.189 | **< 0.001** | | **A < B, C** | **0.121** |
| Periodic limb movements index, nb/h | 6.0 ± 11.0 | 10.0 ± 11.7 | 24.1 ± 26.1 | 50.997 | **< 0.001** | | **A < B < C** | **0.218** |

Data are expressed as mean $\pm$ standard deviation. IH = Idiopathic hypersomnia; med+ = use of antidepressant medication during PSG recordings; med- = no use of antidepressant medication during PSG recordings; REM = Rapid Eye Movement; WASO = Wake After Sleep Onset, min = minutes, nb = number, h = hour.
